# Transforming Agro-Waste Cutin into Sustainable Materials for Biomedical Innovations

**DOI:** 10.3390/polym17060742

**Published:** 2025-03-12

**Authors:** Gianni Pecorini, Martina Tamburriello, Erika Maria Tottoli, Giangiacomo Beretta, Ida Genta, Bice Conti, Rossella Dorati, Rita Nasti

**Affiliations:** 1 Department of Drug Sciences, University of Pavia, Viale Taramelli 12, 27100 Pavia, Italy; 2 Department of Environmental Science and Policy (ESP), Università degli Studi di Milano, Via Celoria 2, 20133 Milan, Italy

**Keywords:** circular economy, bio-based polymers, tomato peels, biomass valorization, agricultural waste, cutin, fatty acids

## Abstract

Agricultural waste derivatives, particularly tomato cutin, a biopolymer found in the cuticular layer of plants, present a promising alternative for the development of sustainable materials in biomedical applications. Cutin, composed primarily of fatty acids and hydroxy acids, exhibits favorable biodegradability, biocompatibility, and hydrophobic properties, positioning it as a viable candidate for applications such as drug delivery systems, wound healing, and tissue engineering. This study investigates the extraction, characterization, and potential biomedical utilization of 10,16-dihydroxy hexadecenoic acid monomer derived from tomato cutin agro-waste. The cytotoxicity of cutin-based materials was evaluated through in vitro assays, demonstrating minimal toxicity and confirming their suitability for biomedical applications. The extraction process was optimized using various solvents, and the molecular characteristics of the extracted monomer were assessed using techniques such as Gel Permeation Chromatography (GPC), Gas Chromatography–Mass Spectroscopy (GC-MS) ^1^H and ^13^C Nuclear Magnetic Resonance (NMR), Fourier Transformed Infrared (FT-IR) spectroscopy, Thermogravimetric Analysis (TGA), and Differential Scanning Calorimetry (DSC). MTT assay was also performed on NHDFs cultured in monomer solutions to assess their cytocompatibility. The findings suggest that cutin-based materials, when processed under environmentally sustainable conditions, offer an effective and biocompatible alternative to conventional synthetic polymers, opening new avenues for the development of sustainable biomedical products.

## 1. Introduction

The events that have characterized the past few years, first among all of them COVID-19, still have a great economic and environmental impact. In fact, in the case of the global pandemic, its management and the beneficial use of innovative technologies has on the one hand, prevented the diffusion of the virus, but on the other hand, increased the use of disposable products and, therefore, increased the volume of plastic waste [1]. According to the United Nations Environment Programme (UNEP), medical facilities worldwide generated an additional 2 kg of healthcare waste per hospital bed, predominantly due to the increased need for protective equipment and disposable medical supplies during COVID-19 [2]. The World Health Organization (WHO) warns that 15% of all healthcare waste is hazardous, posing risks of biological, chemical, or radioactive contamination [3]. As increased waste generation keeps up with air pollution, it is crucial to develop a healthier ecosystem to also offer potential benefits to human health [1]. Addressing these challenges requires a shift from the conventional linear economy to a circular economy, particularly in the health sector. The circular economy prioritizes the reuse, repair, collection, separation, and recycling of materials, thereby transforming production waste and end-of-life products into valuable secondary raw materials [4]. The increasing demand for environmentally friendly materials has driven research into utilizing agro-waste by-products as raw materials to produce biodegradable materials. Among these, 10,16-dihydroxy hexadecenoic acid monomer (10,16-DiHHDA, also indicated in the text as a cutin monomer), a biopolymer mainly composed of long-chain hydroxy fatty acids, found in the epidermal cells of plants, stands out due to its unique properties, including biodegradability, hydrophobicity, and antimicrobial activity. Typically extracted from agro-waste, such as fruit and vegetable peels, the depolymerization of cutin spans, as described in the literature, from more complex and expensive methods to simpler, faster, and more sustainable methods [5]. Cutin has the potential to be transformed into sustainable materials suitable for a variety of applications, including biomedical ones not yet explored in depth in the literature. From the literature on this topic, it is known, for example, that the pharmaceutical sector is exploiting cutin for the synthesis of new renewable co-polymers [6], for the production, in combination with pectin, of comestible films to improve the quality and preservation of food products [7]. Moreover, the development of cutin wraps combined with chitosan produces bioplastics as an environmentally sustainable alternative to conventional plastics used for food packaging [8]. While existing research has explored the general properties and potential applications of cutin, this paper investigates its specific applicability in biomedical fields. Cutin’s unique characteristics suggest it could play a critical role in applications such as drug delivery systems, wound healing, biodegradable implants, and scaffolds for tissue engineering. To this end, tomatoes, whose cuticle dry weight is characterized by cutin in the range of 40 to 85% [9], were used as the extraction source for cutin. Therefore, the proposed paper is in line with the circular economy model; in fact, tomatoes, after potatoes, are one of the most produced and consumed vegetables, with a world production that in 2022 amounted to 186 million tons [10], of which in Italy about 6 million tons [11]. Precisely, of the total world production, 39 million tons are processed annually by food factories that produce, from the main product, 5–30% agricultural waste, which is often used as animal feed or disposed of in landfills [12,13]. In addition, the extraction process has been carried out through the use of supercritical fluids, ensuring a selective, efficient, and more eco-friendly process than widely described methods, such as Soxhlet that usually uses organic solvents (n-hexane or dichloromethane) for cutin extraction [14,15]. The interesting characteristics of carbon dioxide (CO_2_) make it an interesting solvent to use in extraction processes since it is safe for human health and the environment [16], as defined by several organizations such as the Food and Drug Administration (FDA) and the European Food Safety Authority (EFSA) [17,18]. However, this paper is specifically centered on the potential of cutin from agricultural waste for biomedical applications, such as drug delivery systems (DDS), biodegradable implants, and scaffolds for tissue engineering. The extraction purification processes have been carefully designed and developed to ensure high purity, while the characterization of the 10,16-diHHDA cutin monomer has been optimized to meet the stringent requirements for biomedical use, guaranteeing that the final materials are both functional and safe for clinical applications.

## 2. Material and Methods

### 2.1. Materials

All reagents and solvents were purchased from Merck (Merck Life Science S.p.A., Milan, Italy) and used without any further purification if not otherwise specified. The 10,16-dihydroxy hexadecenoic acid (10,16-diHHDA) was isolated from the tomato peel supplied by TomatoFarm s.r.l. (Bettole di Pozzollo, AL, Italy). Thiazolyl blue tetrazolium bromide (MTT) was obtained from Sigma Aldrich (St. Louis, MO, USA). Dulbecco’s phosphate-buffered saline (DPBS) 10× and Tripsyn-EDTA solution 0.25% were purchased by Sigma Aldrich, Milan, Italy. Fetal bovine serum (FBS) was furnished by Immunological Sciences, Rome, Italy. DMEM high glucose w/L-glutamine (w/sodium pyruvate) was obtained from Microgem Laboratory research. Normal human dermal fibroblasts (NHDFs, #LOCC2511) were supplied by Euroclone S.p.A, Pero, Italy. All chemicals which were used were of analytical grade.

### 2.2. Monomers Isolation and Purification

**Figure 1:**
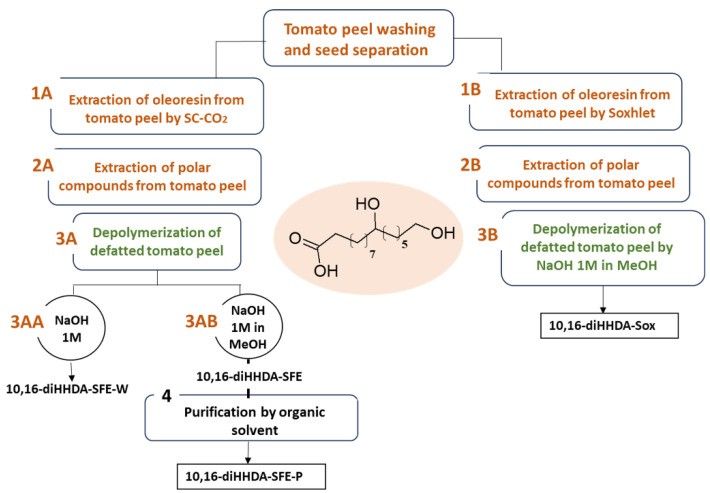
Scheme of 10,16-diHHDA monomer isolation.

After the tomato peels were separated from the seed, they underwent a series of purification steps to recover the pure 10,16-diHHDA (Figure 1). Firstly, the dried peels were micronized and depleted of the characteristic oleoresin, which is rich in carotenoids, by supercritical carbon dioxide extraction (sc-CO_2_), at 400 bar, 60 °C, and a flow rate of 20 kgCO_2_/h (3.7% *w*/*w* of oleoresin) (Figure 1, step 1A). In parallel, almost 20 g of dried and micronized tomato peel was purified from an apolar component by Soxhlet, employing *n*-hexane (1 g: 10 mL, *w*:*v*) for 8 h of dynamic extraction. The mixture was then filtrated on a Buckner, and the organic solvent was evaporated to provide 2.6% of oleoresin (*w*:*w*) (Figure 1, step 1B). On the contrary, the polar fraction of tomato peel, mainly represented by polysaccharides and amino acids, was removed using an aqueous maceration for 12 h at room temperature (Figure 1, 2A and B). The tomato peels were dried to remove the excess water.

**Figure 2:**
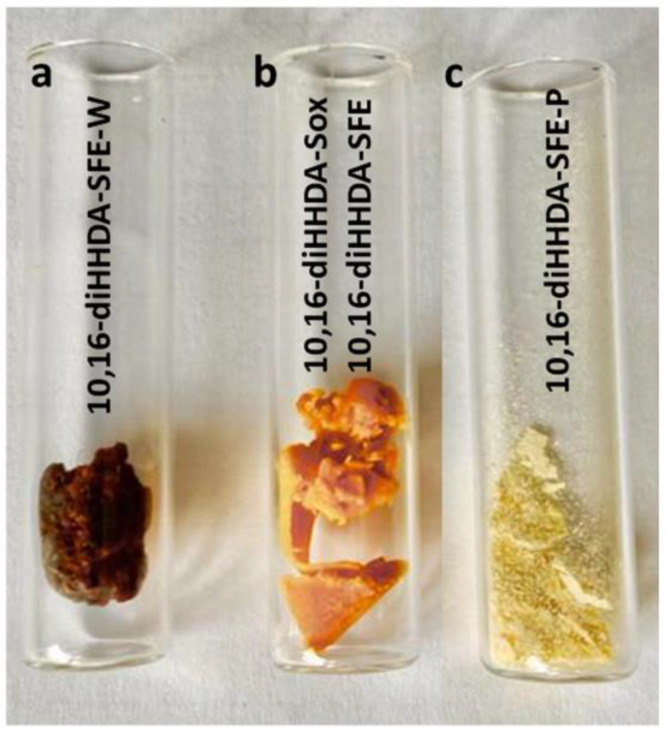
Image of 10,16-diHHDA monomer powder by different depolymerization conditions. From left to right: (**a**) 10,16-diHHDA-SFE-W, (**b**) 10,16-diHHDA-Sox and -SFE, and (**c**) 10,16-diHHDA-SFE-P.

The defatted tomato peels obtained using both methods previously described were subsequently depolymerized with the methanolic sodium solution 1 M for 3 h at reflux. The orange solution was filtered and diluted 3 times with milliQ water for acidification at pH 3 with HCl 37%. At this stage, an orange-reddish precipitation was observed. The suspension was extracted with dichloromethane (DCM) (×3), and the organic phases were collected, dried with sodium sulfate (Na_2_SO_4_), and evaporated through a vacuum to give an orange waxy solid (35–45% *w*/*w* on the whole tomato peel, Figure 1 3AA and 3B and Figure 2). The isolated monomer from the Soxhlet purification process was denominated 10,16-diHHDA-Sox, and that from the Sc-CO_2_ purification process was denominated 10,16-diHHDA-SFE.

To improve the purity of the final product, a further purification step was applied using ethyl acetate (EtOAc) (Figure 1, 4) to precipitate a white waxy powder (Figure 2, 10,16-diHHDA-SFE-P, 60–75%) with a purity of >95%. In this paper, only the data regarding the purified monomer form sc-CO_2_ purification process were showed. The isolation process described has been patented PCT/IB2024/055030 [19].

In the view to optimize a total green process, the depolymerization was also carried out using an aqueous sodium solution (NaOH 1M), maintaining the parameters and steps described before, and avoiding the involvement of organic solvents (Figure 1 3AA). The resulting monomer (10,16-diHHDA-SFE-W) was a reddish and gummy solid (50% *w*/*w* on whole tomato peel, Figure 2).

### 2.3. Physico-Chemical Characterization

#### 2.3.1. Molecular Weight Distribution of Monomers

The analysis of cutin monomer composition, which was obtained by employing different depolymerization and purification conditions, was carried out by employing Gel Permeation Chromatography (GPC). GPC analysis was carried out using a 1260 Infinity GPC apparatus (Agilent Technologies, Santa Clara, CA, USA) equipped with three columns (Plgel 5 µm 500 Å–300 × 7.5 mm, PL aquagel-OH MIXED-H 8 µm 10^3^ Å 300 × 7.5 mm, Phenogel 5 µm 10^4^ Å), a pre-column (Plgel 5 µm 50 × 7.8 mm) and a refractive index detector r (Agilent Technologies 1260 Infinity), and elaborated using a software Cirrus version 3.4 (Agilent Technologies Cernusco s. Naviglio, Milano, Italy). Tetrahydrofuran (THF) was employed as a mobile phase. The samples were dissolved in THF under stirring at an iced bath temperature, and 20 μL were eluted with THF at a 0.8 mL/min flow rate. The calibration curve equation was obtained using monodisperse polystyrene standards with molecular weights ranging from 1430 to 318,500 Da, which is as follows:

LogM = 34.36 − 3.214X^1^ + 0.1174X^2^ − 0.00149X^3^

The curve fitting yielded a coefficient of determination (R^2^) of 0.997128, a residual sum of squares (RSS) of 0.0151467, and a linear correlation coefficient of 1. The molecular weight data were processed to obtain weight average molecular weight (Mw), number-average molecular weight (Mn), and molar dispersity (Đ_M_). The results are presented as the average of three replicate measurements.

#### 2.3.2. Gas-Chromatography Analysis

The purity of 10,16-diHHDA was determined using the Bruker Scion SQ instrument (Bruker, Milan, Italy) gas chromatography, equipped with a ZB-5HT INFERNO capillary column (30 m; 0.25 mm i.d., film thickness 0.25 mm). The oven temperature was initially set at 125 °C (hold time 3 min), with a gradient ranging from 125 to 205 °C (5.0 °C/min, hold 7 min), and 205 to 300 °C (7 °C/min, hold 5 min). The following parameters were then used: injector temperature of 300 °C; column flow of 1.00 mL/min; carrier gas helium of 5.5; ionization energy of 70 eV. The split/splitless ratio was set to 1:30 after 75 s. An aliquot of 10,16-diHHDA was derivatized via the addition of 30 μL of pyridine and 70 μL of N,O-bis(trimethylsilyl)trifluoroacetamide (BSTFA). After 1.5 h of incubation at 62 °C, 500 μL of EtOAc was added and 2.0 μL of the solution was injected into the gas chromatography tandem mass spectroscopy (GC-MS) system. Identification was performed via a comparison of the retention times and fragmentation patterns of the authentic standards when they were available, or via a comparison with those present in the National Institute of Standards and Technology (NIST) Spectral Library (NIST, 2011 vers. 2.0), or via a comparison with those present in the literature [20]. 10,16-diHHDA-TMS R*t *= 34.01 min.

#### 2.3.3. Spectroscopic Characterization of Monomers

Attenuated total reflectance Fourier-transform infrared (ATR-FTIR) spectroscopy. The ATR-FT-IR spectra of 10,16-diHHDA extracted as described in Section 2.2 were recorded in the 4000–400 cm^−1^ range, using an Alpha spectrometer equipped with an ALPHA’s platinum single reflection diamond ATR unit (Bruker Optics, Milan, Italy). The number of scans was *n* 25.

#### 2.3.4. Nuclear Magnetic Resonance Spectroscopy

Proton nuclear magnetic resonance (^1^H-NMR) spectra were recorded on a Bruker Avance 400 spectrometer operating at 400.13 MHz. Proton chemical shifts (δ) are reported in ppm with the solvent reference relative to tetramethyl silane (TMS), which was employed as the internal standard ((CD_3_)_2_SO, δ = 2.54 ppm). The following abbreviations are used to describe spin multiplicity: s represents singlet, d represents doublet, t represents triplet, q represents quartet, m represents multiplet, br represents a broad signal, dd represents doublet–doublet, and td represents triplet–doublet. The coupling constant values are reported in Hz. ^13^C-NMR spectra, which were recorded on a Bruker Avance 500 MHz spectrometer operating at 100.56 MHz, with complete proton decoupling. Carbon chemical shifts (δ) are reported in ppm relative to TMS, with the respective solvent resonance as the internal standard ((CD_3_)_2_SO, δ = 40.45 ppm).

10, 16-dihydroxy hexadecenoic acid: ^1^H-NMR (500 MHz, (CD_3_)_2_SO) = 1.26 (-C*H*_2_, s, 20H), 1.27–1.31 (-C*H*_2_-CHOH-C*H*_2_, m, 4H), 1.39–1.42 (-C*H*_2_-COH, m, 2H), 1.48–1.52 (-C*H*_2_-CH_2_COOH, t, 2H), 2.17–2.20 (-C*H*_2_-COOH, t, 2H), 3.37 (-C*H*OH, s, 1H), 3.38–3.40 (-C*H*_2_OH*,* d, 1H), 4.15 (-CHO*H*, s, 1H), 4.28 (-CH_2_O*H*, s, 1H), 11.91 (-COO*H*, s, 1H) [21].

^13^C-NMR (101 MHz, (CD_3_)_2_SO) = 174.99, 70.18, 61.29, 37.68, 34.15, 33.01, 29.66, 29.64, 29.45, 29.19, 29.03, 26.04, 25.80, 25.72, 24.97 [21].

#### 2.3.5. Thermal Characterization of Monomers

##### Thermogravimetric Analysis

Thermogravimetric analysis (TGA) was performed in inert conditions, heating around 5 mg of sample in a ceramic pan at a heating rate of 10 °C/min in the range 30–600 °C, using a TGA400 instrument (PerkinElmer Inc., Waltham, MA, USA). Degradation temperature was determined as the onset point of the first weight loss.

##### Differential Scanning Calorimetry

Differential scanning calorimetry (DSC) analysis was performed on about 15 mg of the sample using a SC 823e instrument (Mettler Toledo, Columbus, OH, USA). The analysis consisted of one heating cycle at a heating rate of 10 °C/min in the range of 30 to 300 °C under a nitrogen atmosphere using an aluminum pan. Tm was determined as the minimum point of the observed thermal transition.

### 2.4. Solvent Compatibility

The solubility of the cutin monomer was systematically assessed in various solvents, including DCM, THF, ethanol (EtOH), and acetone (Ace). These solvents were selected based on their differing polarities and potential for dissolving 10,16-diHHDA, a monomer with hydrophobic properties. The goal was to evaluate the compatibility of cutin monomer with different solvents and identify the most suitable one for subsequent processing and biomedical applications. Understanding which solvents can dissolve the monomer is vital for its processing (e.g., dissolution, film formation, or composite material fabrication). In total, 30 mg of cutin monomer were added to 1 mL of each solvent, and they were left under magnetic stirring for 24 h. Pictures of the obtained solutions/suspensions were taken soon after solvent addition and after 24 h to qualitatively evaluate the complete dissolution of the monomers or the formation of a solid precipitate.

#### Solubility of Cutin Monomer in Biomedical Solvents

To determine cutin monomer solubility in ethanol, an excess of 10,16-diHHDA-SFE-P (3 g) was added to a round-bottom flask containing 5 mL of EtOH 96% *v*/*v*. The mixture was stirred gently at room temperature for 4 h to ensure thorough dissolution. At predefined time intervals, magnetic stirring was paused to allow the undissolved cutin monomer to precipitate at the bottom of the flask. A 150 μL aliquot of the supernatant was carefully withdrawn, followed by centrifugation at 4 °C at 5000 rpm for 5 min using an Eppendorf 5417R centrifuge (Eppendorf, Milan, Italy). After centrifugation, 10 μL of the supernatant was diluted with 990 μL of THF for further analysis. The cutin monomer concentration in the supernatant was quantified using GPC by correlating the signal intensity with a previously established calibration curve. A calibration curve, established by analyzing 10,16-diHHDA-SFE-P solutions at a concentration range of 1–6 mg/mL (prepared in ethanol and subsequently diluted with THF), was employed to quantify the concentration of the monomer in the supernatant at each time point. The calibration curve equation, y = 2169.4x − 220.54 with a correlation coefficient R^2^ = 0.9703, was applied for cutin monomer quantification. The reported values represent the average of five replicate measurements.

### 2.5. Stability and Storage Conditions of the Monomers

The stability of 10,16-diHHDA-SFE-P, both in its raw material form and in solution, was evaluated to determine the most suitable storage conditions for its use in biomedical applications. As a raw material, cutin monomer was stored under various environmental conditions, including light exposure and temperatures of 4 °C ± 2 °C (refrigerated) and 25 °C ± 2 °C (ambient), to simulate potential storage scenarios. The stability of cutin monomer in an ethanolic solution was assessed by dissolving 10,16-diHHDA-SFE-P in EtOH (96% *v*/*v*) at a concentration of 2.8 mg/mL, and incubating the solutions at room temperature under light for seven days. Changes in the molecular structure and molecular weight of cutin monomer and changes in cutin monomer concentration were monitored over time using GPC. Additionally, the photo-stability of the solutions was evaluated by exposing them to visible light at room temperature. These investigations provided valuable insights into the optimal storage conditions for both raw monomer and its solutions, ensuring that the material maintains its integrity and suitability for further processing into biomedical products such as films, scaffolds, or drug delivery systems.

### 2.6. Cytotoxicity Assay

#### 2.6.1. Cell Culture

The biocompatibility of the purified cutin monomers was determined through an MTT assay. The analyzed monomers were 10,16-diHHDA-Sox, and the sc-CO_2_ extracted cutin depolymerized through methanolysis, carried out at different temperatures (10,16-diHHDA-SFE-WARM and 10,16-diHHDA-SFE-RT), 10,16-diHHDA-SFE-P, and 10,16-diHHDA-SFE-W. The assay was carried out using NHDFs derived from primary culture (Dermal Fibroblast Adult, amp 500 kCells, Lonza, Milan, Italy). Cells were resuspended in DMEM containing 10% *v*/*v* of FBS, 1% *v*/*v* of antibiotic mixture (100 µg/mL penicillin, 100 µg/mL streptomycin), and 1% *v*/*v* glutamine. Fibroblasts were thawed and immediately suspended in DMEM; the suspension was centrifuged for 5 min at 1500 rpm and 4 °C (Sorvall TC and Centrifuge, Thermofisher, Waltham, MA, USA); the obtained pellet was resuspended in fresh media, transferred in T75 flask, and incubated at 37 °C and 5% CO_2_. During the proliferation, the morphology and growth of cells were evaluated daily with a reverse optic microscope (LEICA DM 13000B, Leica Microsystems, Milan, Italy).

#### 2.6.2. MTT Assay

An MTT assay was carried out according to ISO 10993-9 [22]. Cutin monomer solutions were prepared by dissolving cutin monomers into EtOH obtaining a stock solution and dilution using a cell medium until achieving a final concentration of 0.75 mg/mL. The analyzed monomers were 10,16-diHHDA-Sox, 10,16-diHHDA-SFE-WARM, 10,16-diHHDA-SFE-RT, 10,16-diHHDA-SFE-P, and 10,16-diHHDA-SFE-W. The working solution was finally sterile-filtered before use. After reaching confluence, the cells were trypsinized, resuspended in a fresh medium, and seeded in a 96-well plate at a cell density of 1 × 10^4^ cells/well. After 24 h from the seeding, the cell medium was replaced with a working solution. At selected time points, (24 h and 48 h from the medium replacement) the cut solution was replaced with a fresh medium and 25 μL of a MTT solution 5 mg/mL was added to the well. The cells were then incubated at 37 °C, using 5% CO_2_ for 2.5 h. The absorbance of each well was measured at 570 nm using a microplate reader, Hipo MPP-96 (SIA, Biosan, Riga, Latvia). Three replicates of each sample were analyzed. The cells cultured in DMEM were employed as positive controls, while cells treated with dimethyl sulfoxide (DMSO) were used as negative controls.

#### 2.6.3. Morphological Characterization

The morphological characterization of fibroblast cells exposed to 10,16-diHHDA-Sox, 10,16-diHHDA-SFE-P, and 10,16-diHHDA-SFE-W solutions was conducted using a light microscope to visually assess any potential cytotoxic effects. Following the MTT assay, cell morphology was observed at 24 h and 48 h time points to identify changes in cell shape, attachment, or confluence, which could indicate cytotoxicity or adverse effects from the material. A LEICA DMIL LED Fluo optical microscope (Leica Microsystems, Milan, Italy) was employed for the morphological analysis, allowing for the detailed observation of any alterations in cell morphology, such as detachment, swelling, or changes in cell density. These observations were then compared to the control groups to assess potential cytotoxic effects. Cells cultured in standard DMEM medium served as the positive control, while cells exposed to DMSO acted as the negative control, to ensure that any observed changes were due to the interaction with the cutin material.

#### 2.6.4. Osmolarity

The osmolarity of 10,16-diHHDA-Sox, and 10,16-diHHDA-SFE-P solutions were quantified utilizing a freezing point osmometer (Freezing Point Osmometer, FPO-2000, Bievopeak, Jinan, China). Monomer solutions were prepared by dissolving monomers in 96% (*v*/*v*) EtOH, followed by dilution with DMEM supplemented with 10% FBS to achieve the target concentrations. Prior to measurements, all solutions were filtered through cellulose membrane filters with a pore size of 0.22 μm.

Subsequently, 1 mL aliquots of each solution were transferred into 1.5 mL plastic tubes for an osmolarity assessment. A control solution, representing the pure solvent, was similarly prepared by mixing DMEM containing 10% FBS with 96% (*v*/*v*) ethanol in the same ratio used for monomer solutions. The samples were analyzed by placing them in the osmometer probe, which determined the freezing point of each solution and calculated the freezing point depression relative to the pure solvent. Based on the freezing point depression data, the osmometer directly computed the osmolarity of the solutions (mOsm/L).

### 2.7. Statistical Analysis

The data were presented as the mean ± standard deviation (*n* = 3). To analyze differences in the mean values between experimental groups, a two-way ANOVA (analysis of variance) was performed, followed by Tukey’s multiple comparison test, using GraphPad Prism 7.0 software. The significance levels were defined as * *p* < 0.05, ** *p* < 0.01, *** *p* < 0.001, and **** *p* < 0.0001.

## 3. Results and Discussion

### 3.1. Monomers Isolation, Purification and Characterization

The isolation of 10,16-diHHDA from tomato cutin has attracted the attention of several research groups over the last decade, particularly for coating applications and the development of new biodegradable plastics, according to the pioneering work of Osman et al. [23]. For these applications, a mixture containing the 10,16-diHHA monomer, related isomers, cuticular fatty acids, and other components has been blended with other polymers to achieve satisfactory results in coating and bioplastic applications [5,9]. In contrast, the use of the cutin monomer in the pharmaceutical field requires strict and well-defined chemical characterization to meet regulatory requirements and ensure the reproducibility of large-scale processes. The papers in the literature that use the monomer for formulations with other pharmaceutical components typically employ the synthetic analog.

To achieve this goal, a tailored process for isolating 10,16-diHHDA was developed [19]. The purification of the tomato peel is crucial in obtaining the pure monomer, and this paper presents an innovative approach designed to minimize interference and sub-products during the depolymerization reaction. Supercritical fluid extraction was selected as a sustainable alternative to solvent-based extraction methods (e.g., Soxhlet), leveraging its ability to selectively extract apolar compounds by adjusting key parameters such as pressure and temperature. Differently from the Soxhlet, the use of SFE guarantees the absence of organic solvent and a defined chemical composition of the oleoresin. Indeed, the obtained oleoresin represents a further secondary raw material that could be repurposed for applications in materials and cosmetics as a natural colorant. By implementing this procedure before the depolymerization step, it helped reduce the interference from the apolar components of the natural matrix, thereby preventing the formation of emulsions and minimizing the presence of difficult-to-remove sub-products. The set-up procedures (Figure 1, steps 3 and 3B) yielded an orange waxy solid (Figure 2, 3AB and 3B), and were easy to manage and characterize. Both monomers (10,16-diHHDA-Sox and -SPE) did not show significant differences in terms of chemical composition, except for the presence of cuticula fatty acid in 10,16-diHHDA-Sox crude (15%).

**Figure 3:**
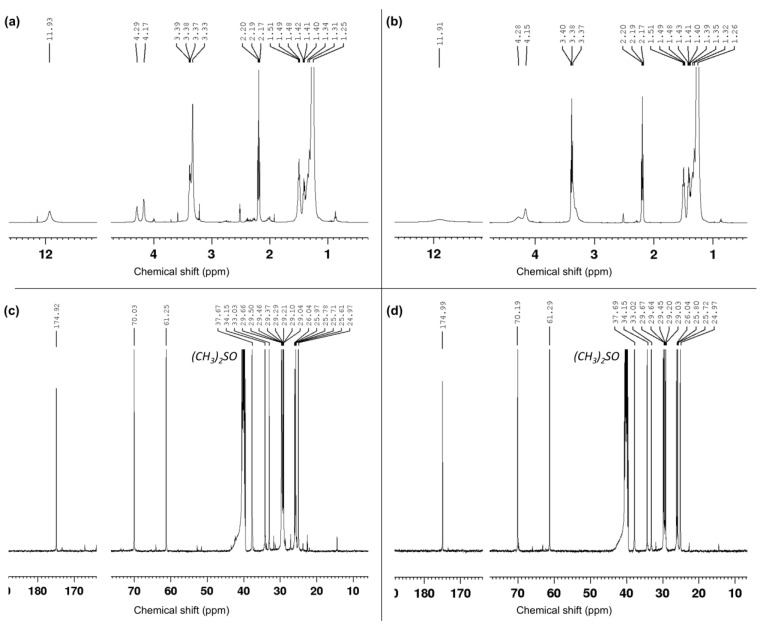
^1^H-NMR of (**a**) 10,16-diHHDA-SFE and (**b**) 10,16-diHHDA-SFE-P; ^13^C-NMR of (**c**) 10,16-diHHDA-SFE and (**d**) 10,16-diHHDA-SFE-P acquired in *(CD*_3_*)*_2_*SO*.

**Figure 4:**
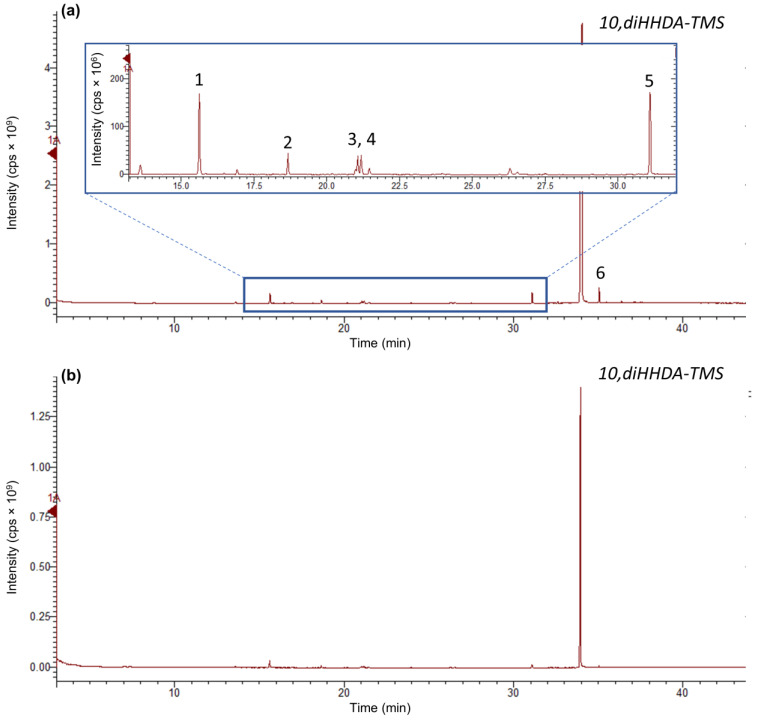
Chromatographic profile of (**a**) 10,16-diHHDA-SFE (numbers in the figure are referred to 1—cinnamic acid, p-(trimethylsiloxy)-, methyl ester; 2—isomer of cinnamic acid, p-(trimethylsiloxy)-, methyl ester; 3—hexadecanoic acid (trimethylsiloxy); 4—azelaic acid (trimethylsiloxy); 5—(trimethylsiloxy)isomer of 10,16-diHHDA; 6—(trimethylsiloxy) of 10,18-diHHDA) and (**b**) 10,16-diHHDA-SFE-P.

**Figure 5:**
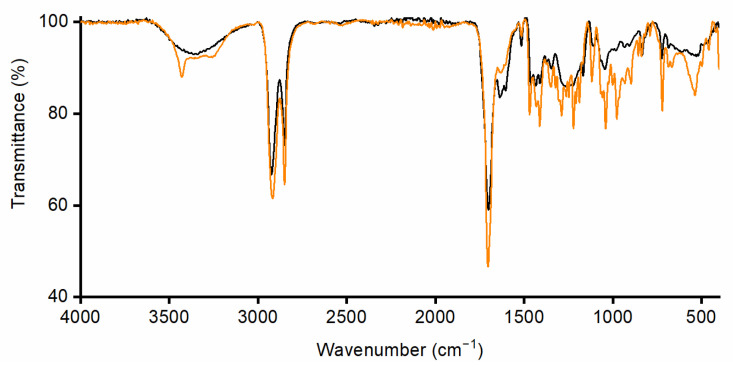
Superimposition of ATR-FTIR spectra of 10,16-diHHDA-SFE (black) and 10,16-diHHDA-SFE-P (orange).

As shown in the chromatogram and in the ^1^H-NMR spectrum (Figure 3a and Figure 4a), the 10,16-diHHDA was the main component, followed by structural isomer and cuticular fatty acids together with different compounds at lower concentrations, as evidenced by the zoomed region in Figure 4a. The compounds present in the zoomed region of chromatogram (a) were identified as follows: 1—cinnamic acid, p-(trimethylsiloxy)-, methyl ester; 2—isomer of cinnamic acid, p-(trimethylsiloxy)-, methyl ester; 3—hexadecanoic acid (trimethylsiloxy); 4—azelaic acid (trimethylsiloxy); 5—(trimethylsiloxy)isomer of 10,16-diHHDA; 6—(trimethylsiloxy) of10,18-diHHDA. The treatment of the crude with an organic solvent removed the other components, and, in particular, the carotenoids degraded subproducts (Figure 3b and Figure 4b) that confer the orange color to the waxy solid. The reduction in carotenoid degradation products is clearly visible via ATR-IR analysis of 10,16-diHHDA-SFE-P (Figure 5). The ATR-IR spectra showed the characteristics bands of cutin: the stretching of hydroxyl groups (3350 cm^−1^), methylene (CH_2_ 2920 cm^−1^), methyne (CH) 2850 cm^−1^, and carbonyl groups (C=O 1729 cm^−1^). Moreover, it was possible to detect the signal relative to the scissoring and the rocking of CH_2_ groups (1467 and 718 cm^−1^, respectively) [24]. The main difference between the two spectra associated with the monomer treatment with EtOAc is the stretching of the alkene decrease after the organic solvent treatment (1636.13 cm^−1^) (Figure 5) [25] and the presence of a new peak at 3433 cm^−1^, which may be associated with the presence of crystallization water, which usually gives a sharp signal in this range of wavelengths [26].

In the optics of the design, for the whole green process to recover the monomer, the depolymerization reaction was attempted in a basic aqueous solution as well, applying the same procedure described mainly in the literature. However, the reaction led to a monomer (10,16-diHHDA-SFE-W), which was hard to manage, with a high texture variability along the repeated reactions. Its texture was gummy and sticky, which did not allow for the full chemical characterization.

**Figure 6:**
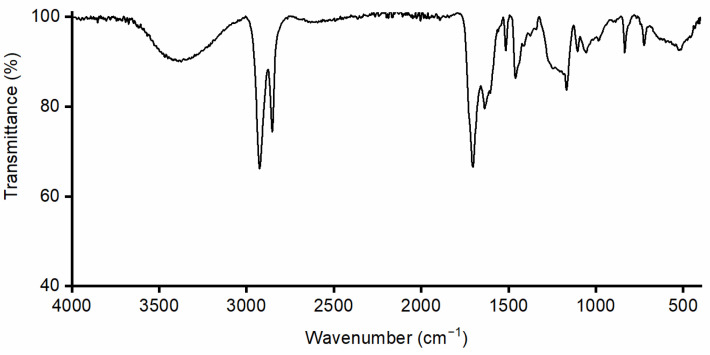
ATR-FTIR spectrum of 10,16-diHHDA-SFE-W.

This was probably due to the water and polar components incorporated during the precipitation of the monomer at pH 3.5, as showed by the ATR-IR profile via the broad signal at 3300 cm^−1^, which is usually associated with the stretching of hydroxyl groups (Figure 6). We tried to remove the water by lyophilization with unsuccessful results. Moreover, the purification step (Figure 1 step 4) was attempted as well on the 10,16-diHHDA-SFE-W, with the formation of a compact pellet, leading to an unsuccessful result.

### 3.2. Physico-Chemical Characterization

#### 3.2.1. Molecular Weight Distribution of Monomers

**Figure 7:**
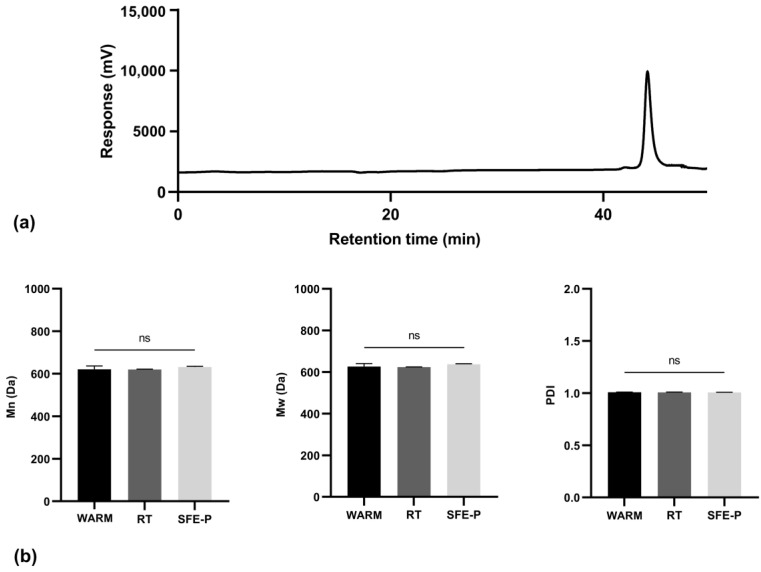
(**a**) GPC chromatogram of 10,16-diHHDASFE-P; (**b**) confrontation of Mn Mw, and PDI of 10,16-diHHDA obtained at different conditions.

GPC analysis was performed on the purified cutin monomers to validate the depolymerization reaction, confirm that the sample consists of a single molecular weight distribution, and ensure the absence of impurities. A representative chromatogram of the cutin monomers, obtained through SFE extraction following our patented procedure is shown in Figure 7.

The chromatographic analysis revealed a single peak with a retention time of 44 min, confirming the successful depolymerization of cutin and indicating a significant reduction in its molecular weight. Mn was determined to be 632 ± 3 Da, while the Mw was 637 ± 3 Da, yielding a Đ_M_ of 1.006 ± 0.002. This low Đ_M_ suggests a uniform molecular weight distribution in the depolymerized product. The literature reports suggest that cutin’s molecular weight can vary widely, often within the range of 500 to 9000 Da before depolymerization, with values influenced by species, environmental conditions, and the degree of cross-linking. After depolymerization, the molecular weight is typically much lower, reflecting the breakdown of the polymer into its monomeric or oligomeric forms [5]. Moreover, the literature reports indicate that cutin’s molecular weight inherently varies, which is attributed to its complex structure, monomer composition, and the degree of cross-linking, which can fluctuate based on plant species and environmental conditions [5,27].

The observed Đ_M_ of 1.02 indicates a highly uniform molecular weight distribution in the depolymerized product, suggesting that the depolymerization reaction was effective and yielded a product with minimal variability in molecular size. This low Đ_M_ is consistent with the effective depolymerization reported in the literature, where well-controlled depolymerization processes often yield products with narrow molecular weight distributions close to 1.0 [5,28].

The presence of a single chromatographic peak further verified the absence of compounds with differing molecular weights, implying the effective removal of any non- or partially depolymerized monomers and impurities during the purification process. The literature indicates that successful depolymerization should ideally yield a single peak if impurities and partially depolymerized chains are effectively removed [5]. Moreover, the low standard deviations observed underscores the precision and consistency of the measurements. Comparative batch-to-batch analyses confirmed the reproducibility and robustness of both the depolymerization and purification protocols, highlighting the reliability of these processes. Consistency across batches is crucial in polymer depolymerization studies to confirm that the process can be reliably replicated [5,28].

Chromatographic analysis of 10,16-diHHDA-SFE-WARM, -RT, -P revealed no statistically significant differences in the Mn, Mw or Đ_M_ parameters across the samples, as shown in Figure 7b.

#### 3.2.2. Thermal Characterization of Monomers

Thermal characterization was carried out through TGA and DSC carried out only on 10,16-diHHDA-SFE-P since it is the one selected for future studies according to the results of the biological characterization (see Section 3.6).

**Figure 8:**
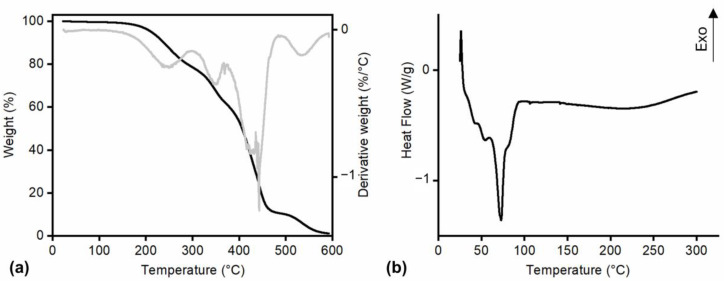
(**a**) TGA of 10,16-diHHDA-SFE-P (black curve weight vs. temperature, gray curve first derivative of weight vs. temperature curve); (**b**) DSC analysis 10,16-diHHDA-SFE-P.

Considering the literature on cutin’s thermal stability, the TGA presented in Figure 8a supports the known behavior of cutin as a relatively stable biopolymer under moderate temperatures. Cutin has been reported to exhibit stability up to around 150 °C, which is consistent with the observed retention of 100% weight in the sample up to this temperature. Furthermore, the onset of substantial degradation at approximately 300 °C, where a 20% weight loss occurs, is consistent with previous studies on cutin’s decomposition profile, confirming its characteristic thermal stability at elevated temperatures [29,30].

The results of the DSC analysis on 10,16-diHHDA-SFE-P showed an endothermic peak with three different shoulders associated with the melting of sample crystals (Tm 72 °C) Figure 8b [27]. However, further temperature increases did not result in any observable phase transitions related to crystallization or melting in the temperature range of 100–300 °C with the only exception of an exothermic peak between 250 and 300 °C. This peak, according to TGA, may be attributed to sample thermal degradation. The crystallinity of the sample may be the reason for the slow dissolution of 10,16-diHHDA-SFE-P in EtOH as highlighted in Section 3.4. It is not possible to observe the evaporation of the crystallization water evidenced by the FT-IR analysis probably because it is covered by the melting of the crystals.

### 3.3. Solvent Compatibility

**Figure 9:**
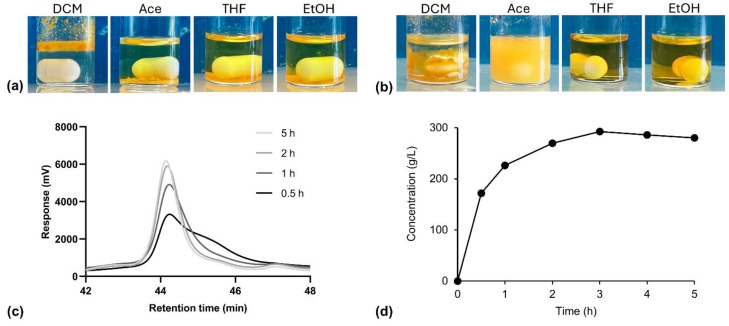
Compatibility of 10,16-diHHDA-SFE-P with DCM, Ace, THF, and EtOH (**a**) after solvent addition; (**b**) 24 h from solvent addition; dissolution kinetics of cutin in EtOH (**c**) cutin signal at different dissolution times (0.5–5 h) obtained through GPC analysis; (**d**) cutin concentration vs. time.

The solubility of 10,16-diHHDA-SFE-P was systematically assessed across a selection of solvents with varying polarities, including dichloromethane (DCM), acetone (Ace), tetrahydrofuran (THF), and ethanol (EtOH), to determine solvent compatibility for potential biomedical applications. The four different solvents were added to monomer powder and pictures of the samples soon after the solvent addition and after 24 h from the solvent addition are reported in Figure 9a,b.

Given the low water solubility of fatty acids, these solvents were chosen based on their polarity profiles and effectiveness in dissolving similar compounds. Each solvent was evaluated to identify its suitability for processing cutin monomer in applications such as dissolution, film formation, and composite material fabrication. THF and EtOH demonstrated favorable solubility properties, making it particularly noteworthy; however, differently from THF, as a Class 3 solvent with low toxicity under ICH Q3C (R8) guidelines, EtOH is an ideal candidate for biomedical uses due to its safety profile. The results confirmed that EtOH supports cutin monomer solubilization effectively, providing an optimal solvent environment for further formulation development aimed at biomedical applications. Ace is also a Class 3 solvent, and even though it did not completely dissolve the sample, it allowed for the formation of a stable and homogeneous suspension, which may possibly be employed as an alternative way of cutin monomer processing, employing a novel and more eco-friendly solvent than the conventionally employed. DCM was the worst solvent among the tested ones, and did not allow for cutin monomer dissolution or the formation of a suspension.

### 3.4. Solubility of Cutin in Biomedical Solvents

The 10,16-diHHDA-SFE-P solubility in EtOH was evaluated, as EtOH is classified as a Class 3 solvent [31] and is considered a low-toxicity solvent with minimal human health risks, making it a suitable candidate for the development of medical devices or DDS. The cutin monomer was added to EtOH under supersaturation conditions at room temperature and stirred magnetically for 5 h. At predefined time intervals, the monomer concentration in the supernatant was analyzed via GPC. The dissolution kinetics of the cutin are illustrated in Figure 9c,d.

The data revealed a progressive increase in 10,16-diHHDA-SFE-P concentration over the 5 h test period. Initially, a rapid burst of monomer dissolution was observed during the first 30 min, followed by a sustained increase in concentration that plateaued after approximately 3 h. The plateau concentration of 286 ± 6 g/L represents the solubility of the monomer in EtOH at room temperature. The solubility of 10,16-diHHDA-SFE-P is influenced by both its inherent amorphous nature and its hydrophobicity. Amorphous materials, in general, tend to be more soluble in polar solvents like ethanol due to their lack of crystalline structure, which allows for easier molecular interaction with the solvent. However, the monomer’s hydrophobic properties complicate this behavior, as hydrophobic materials are typically less soluble in polar solvents like ethanol, leading to slower dissolution rates and potential aggregation in aqueous solution. Although 10,16-diHHDA-SFE-P monomer’s amorphous structure suggests it should be more soluble in ethanol compared to crystalline substances, its hydrophobicity counteracts this effect by hindering its interaction with the solvent [5,8].

### 3.5. Stability and Storage Conditions of the Monomers

**Figure 10:**
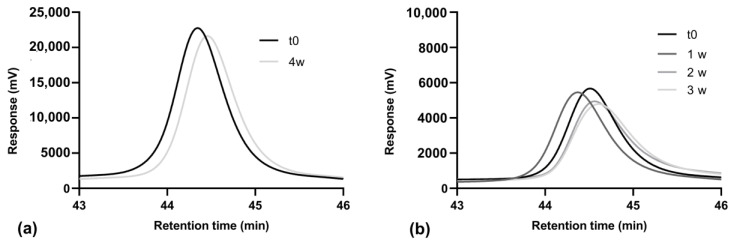
(**a**) GPC chromatograms of 10,16-diHHDA-SFE-P at t0 and after four weeks of storage at ~25 °C; (**b**) GPC chromatograms of cutin solutions in EtOH after different dissolution times.

At 4 °C ± 2 °C, cutin monomers show minimal degradation, retaining their molecular integrity over extended periods. Studies have demonstrated that at lower temperatures, enzymatic activity, oxidation, and unwanted polymerization are significantly slowed down. Bio-based molecules like cutin monomer, when stored in cool conditions, typically retain their solubility and molecular weight, ensuring that they remain effective for use in applications such as medical devices and controlled-release formulations. In this work, the stability of 10,16-diHHDA-SFE-P was tested at ambient temperature (~25 °C) under light for 4 weeks. When stored at these conditions, cutin monomers, being hydrophobic and prone to oxidation, can undergo slow molecular weight degradation and slight oxidation, which could impact their solubility and bioactivity. Cutin monomer samples (2 h, 1 week, 3 weeks) were analyzed through GPC analysis, which revealed that at the beginning of the experiment (t0, after 2 h of monomer addition to EtOH) cutin showed a Mn of 633 ± 3 Da, and a Mw of 637 ± 3 Da with a Đ_M_ of 1.006 ± 0.002, which were not significantly different from the same data obtained after four weeks of monomer storage 649 ± 15 Da, 652 ± 14 Da, and 1.006 ± 0.001, Figure 10a.

Exposure to solvents such as EtOH, which are frequently used to dissolve cutin monomers for biomedical purposes, did not significantly alter the stability of these monomers under typical storage conditions. The stability was demonstrated by monitoring cutin molecular weight and its concentration at different times. After 2 h of magnetic stirring, cutin presented a Mn of 630 ± 7 Da, a Mw of 634 ± 7 Da, and a Đ_M_ of 1.006 ± 0.000 that were not significantly different from the values obtained after 1 w of magnetic stirring: Mn of 638 ± 3 Da, a Mw of 644 ± 2 Da, and a Đ_M_ of 1.009 ± 0.002, Figure 10b. Signal height was employed for the evaluation of cutin monomer concentrations at the tested times, and it concluded that at t0, solution concentration was 2.7 ± 0.2 mg/mL, and after 1 w, it was 2.6 ± 0.1 mg/mL, with no significant differences between the two values, confirming that the cutin monomer did not degrade to form compounds undetectable using GPC analysis. After two weeks, both cutin monomer Mn and Mw did not significantly change (603 ± 12 Da and 611 ± 12 Da, respectively), but a partial degradation of the cutin monomer was confirmed via the measurement of its concentration, which significantly decreased to 2.4 ± 0.1 mg/mL. The chromatograms did not show signals related to different chemical compounds, probably because the molecular weight of the degradation products was too low to be detected by GPC analysis. Similar values were obtained after 3 w (Mn: 602 ± 29 Da, Mw: 612 ± 27 Da, and cutin monomer concentration to 2.4 ± 0.1 mg/mL), with no significant differences, with respect to the values obtained for the 2 w. On the contrary, these 3 w results were significantly lower than the values obtained at t0.

### 3.6. Cytotoxicity Assessment

**Figure 11:**
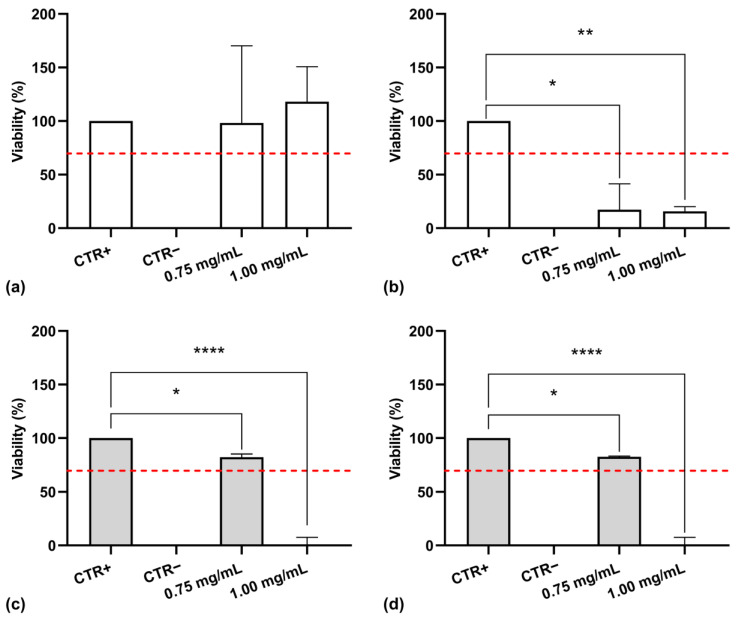
MTT assay performed on 10,16-diHHDA-Sox at different concentrations with treatment durations of (**a**) 24 and (**b**) 48 h; and on 10,16-diHHDA-SFE-P at different concentrations with treatment durations of (**c**) 24 and (**d**) 48 h. Data are reported as mean ± standard deviation (*n* = 3). Statistically significant differences in cell viability are indicated by black lines, with significance levels represented as follows: * *p* < 0.05, ** *p* < 0.01, **** *p* < 0.0001. Red dashed line indicates the 70% of cell viability.

The in vitro cytotoxicity assessment of cutin monomers that were isolated and processed by different extraction methods was essential to determine their suitability for biomedical applications. In accordance with ISO 10993-5 standards [21] for biocompatibility testing, cytotoxicity was evaluated using the MTT assay on NHDF. The cells were exposed to cutin solutions at varying concentrations (0.75 and 1.00 mg/mL) for 24 and 48 h, after which cell viability was measured. As shown in Figure 11a,b, 10,16-diHHDA-Sox for both concentrations showed cell viability values higher than 70% after 24 h of incubation. Nevertheless, the same solutions exhibited cytotoxic effects after 48 h of incubation, leading to a marked decrease in cell viability, making 10,16-diHHDA-Sox unsuitable for the development of biomedical devices.

Figure 11c,d contains the results obtained from the MTT assay, which was carried out on the solutions of 10,16-diHHDA-SFE-P at 0.75 and 1.00 mg/mL. The assay showed that the solution at 0.75 mg/mL showed cell viability values higher than 70% at both the investigated times, while the solution at a concentration of 1.00 mg/mL showed a significantly reduced cell viability after 24 and 48 h of incubation.

**Figure 12:**
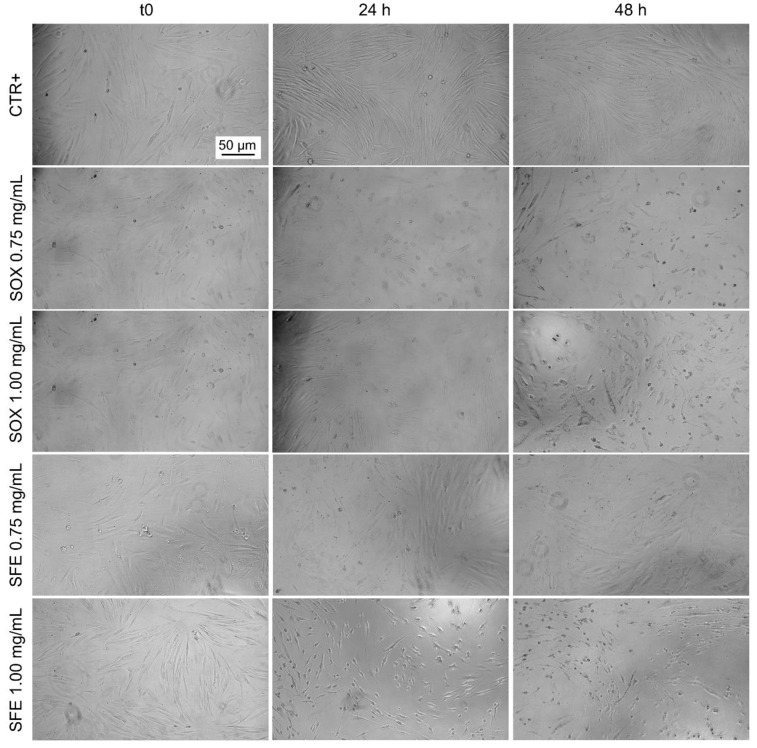
Optical microscopy images (10× magnification) of cells treated with 10,16-diHHDA-Sox (SOX) and 10,16-diHHDA-SFE-P (SFE) for 24 and 48 h at a concentration of 0.75 and 1.00 mg/mL.

A morphological analysis was carried out on cells treated with both typologies of cutin monomer solutions at the tested concentrations after 24 and 48 h of incubation. In Figure 12, these are depicted as the obtained micrographs.

Morphological analysis revealed significant changes in cell morphology, including cell shrinkage and membrane distortion, suggesting a dose-dependent response to the Soxhlet-extracted cutin and the highest concentration of 10,16-diHHDA-SFE-P monomer.

The Soxhlet extraction method often yields a mixture containing not only cutin but also a variety of co-extracted substances, including impurities and other waxy components, which are also present as sub-products in crude monomers after methanolysis.. When dissolved in EtOH and subsequently diluted in DMEM, these additional solutes significantly elevate osmolarity beyond physiological levels, rendering the solution unsuitable for biomedical applications. Osmolarity measurements of the 10,16-diHHDA-Sox- monomer solution showed a value of approximately 864 ± 89 mOsm/L, which is significantly higher than the physiological osmolarity range of blood (275–290 mOsm/L). This osmolarity is also much higher than that of a 0.9% (*w*/*v*) NaCl solution (290 ± 10 mOsm/L) and the standard osmolarity of DMEM (333 ± 22 mOsm/L). The increased osmolarity suggests that the Soxhlet extraction introduces excess solutes, potentially resulting in a hypertonic solution that is unsuitable for biological applications. This elevated osmolarity can be likely attributed to the presence of excess solutes, including co-extracted impurities and waxy components, which are inherent to the Soxhlet extraction process. These additional solutes increase the total solute concentration when the solution is diluted in DMEM, thereby rendering the medium hypertonic. Hypertonic environments can induce osmotic stress in cells, leading to cellular shrinkage, damage, and compromised viability, as previously observed in cell culture studies. Such conditions underscore the importance of refining extraction methods or further purification steps to mitigate the osmotic effects in biological applications. Furthermore, osmolarity measurements of the 10,16-diHHDA-SFE-P monomer solution revealed a value of approximately 450 ± 33 mOsm/L. While this value exceeds the physiological osmolarity range, it remains within a compatible range for cell culture conditions. These findings align with previous studies suggesting that solutions derived from natural polymers like cutin can exhibit osmolarity levels that are compatible with physiological conditions.

**Figure 13:**
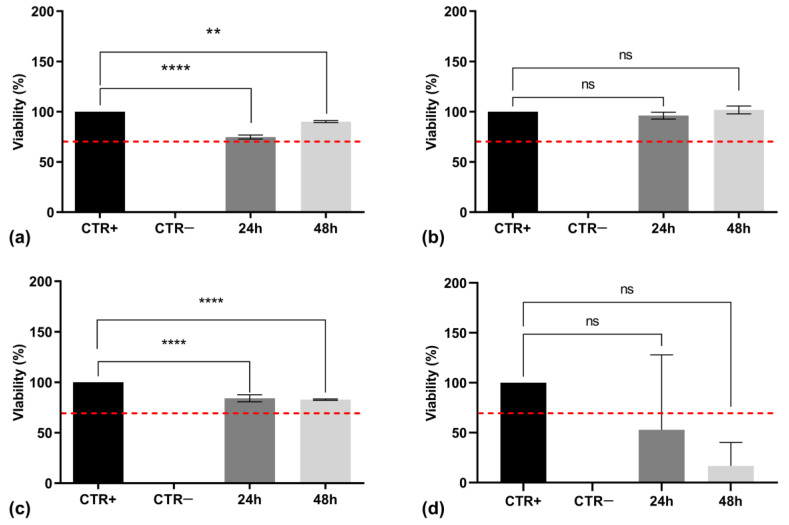
MTT assay carried out on (**a**) 10,16-diHHDA-SFE-WARM; (**b**) 10,16-diHHDA-SFE-RT; (**c**) 10,16-diHHDA-SFE—P; (**d**) 10,16-diHHDA-SFE-W. Data are reported as mean ± standard deviation (n = 3). Statistically significant differences in cell viability are indicated by black lines, with significance levels represented as follows: ** *p* < 0.01, **** *p* < 0.0001. Red dashed line indicates the 70% of cell viability.

Figure 13a–c presents the viability data for 10,16-diHHDA-SFE—: —WARM (Figure 13a), —RT (Figure 13b), and —P (Figure 13c). In Figure 13d, cytotoxicity data are shown for 10,16-diHHDA-SFE-W. These comparative results offer valuable insights into the biocompatibility of different cutin monomers, which are crucial for their potential medical applications.

The MTT assay results demonstrated that all monomer samples obtained through different processes exhibited a cell viability greater than 70%, a threshold defined by ISO 10993-9 (Figure 13a–c). These results further confirm the previous results by showing good biocompatibility of the cutin monomers isolated through SFE processes and depolymerized-employing methanol, and it demonstrates that different depolymerization conditions do not affect its biocompatibility. This suggests that the SFE process is effective as a purification for monomer depolymerization, making it a viable candidate for the development of medical devices.

**Figure 14:**
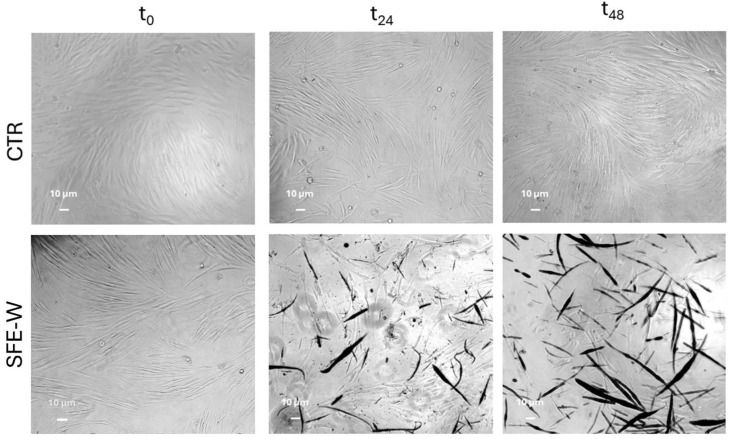
Optical microscopy images (10× magnification) of NHDF treated with 10,16-diHHDA-SFE-W (SFE-W), for 48 h (0.75 mg/mL).

Conversely, 10,16-diHHDA-SFE-W monomer depolymerization in an aqueous environment showed a decrease in cell viability to below 70% after 48 h (Figure 13d). This reduced viability could be attributed to the formation of a precipitate on the surface of the cells during incubation, potentially due to the low solubility of the monomers in the culture medium, (Figure 14). Precipitate formation, especially due to the low solubility of compounds, can interfere with the colorimetric measurement of cell viability. In the context of the MTT assay, formazan crystals are typically produced within viable cells. However, if there is an excess of insoluble materials, such as the precipitates formed from poorly soluble monomers, they can prevent proper solubilization of the formazan product, thereby affecting the accuracy of absorbance reading [32]. Thus, the interference caused by precipitates, in combination with morphological changes in treated cells, supports the hypothesis that poor solubility or precipitate formation could explain the reduced viability observed with the 10,16-diHHDA-SFE-W samples.

Figure 14 presents the morphological analysis of cells treated with a 0.75 mg/mL aqueous solution of 10,16-diHHDA-SFE-W, as assessed at time zero and after 24 and 48 h. Untreated cells were used as positive controls. The analysis revealed the formation of noticeable precipitates, which aggregated over time, indicating the precipitation of cutin in the culture medium. This phenomenon suggests that the low solubility of the 10,16-diHHDA-SFE-W monomers in water led to the aggregation of the monomers on the cell surface, potentially affecting both the cells’ morphology and the accuracy of subsequent viability assessments.

To conclude, the SFE process likely provides a more suitable osmolarity for cell culture applications precisely because of its selectivity and purity, which minimizes the osmotic load from impurities. This makes SFE a preferable choice over Soxhlet extraction for preparing cutin extracts that are intended for use in cell culture environments. Moreover, the 10,16-diHHDA-SFE-P has been selected as the most suitable cutin extract for biomedical applications.

## 4. Conclusions

Among the numerous papers on the conversion of tomato peel into sustainable polymers for innovative new materials, this paper demonstrates the feasibility of introducing 10,16-diHHDA into the biomedical field.

In compliance with the strict regulations governing this area, the study focused on defining the optimal conditions for obtaining a raw material with high purity and precise chemical characterization, which is essential for future medical formulations. This is a key parameter in ensuring proper biological characterization. The introduction of green methodologies into the conventional process for obtaining the 10,16-diHHDA monomer guarantees the reduction in interfering by-products and the isolation of a pure compound (>92%). A process completely free of organic solvents could not be designed based on the results obtained with 10,16-diHHDA-W, right now. Organic solvents such as dichloromethane and ethyl acetate solubilize the by-products, interfering with the purity of the monomer, an essential characteristic for introducing the material into the biomedical and pharmaceutical fields. The study concludes that SFE is a suitable method for isolating cutin monomers from tomato peels compared to traditional Soxhlet extraction. SFE yields purer extracts with reduced osmolarity, a valuable characteristic for biomedical applications. Additionally, among the isolated cutin monomers, 10,16-diHHDA-SFE-P stands out due to its favorable properties, including biodegradability, biocompatibility, and low cytotoxicity. These attributes make the monomer a promising candidate for various biomedical applications, such as drug delivery systems, wound healing, and tissue engineering. Most importantly, this study demonstrates the potential to valorize agri-food waste, such as tomato peel, into valuable biomedical materials for advanced biomedical challenges.

## Data Availability

No additional data available.
